# Enchondroma of the Big Toe for Twenty Years

**Published:** 1860-04

**Authors:** 


*Enchondroma of the big toe for twenty years.*—The growth of enchondromatous or cartilagincus tumour is usually slow; but the period of time varies much, from a few to many years. They are common enough about the hands, but not so much so on the feet.

On the 22nd ult., Mr. Hilton removed from a woman a tumour of this kind, as large as a walnut, which had been slowly growing on the inner surface of the left great toe for the period of twenty years. It recurred after the removal, at that time, of a probably similar tumour, and on the present occasion did not involve the phalanx. She had experienced no pain, and her general health was good; but the position of the growth now and then caused much inconvenience. It proved to be pure enchondroma, without any mixture of bony material, and since the operation the patient has gone on well without a bad symptom. In certain instances similar tumours have increased very rapidly within a few months, but, as a rule, their growth is tardy.—*London Lancet.*

